# Target Product Profile Analysis of COVID-19 Vaccines in Phase III Clinical Trials and Beyond: An Early 2021 Perspective

**DOI:** 10.3390/v13030418

**Published:** 2021-03-05

**Authors:** Colin D. Funk, Craig Laferrière, Ali Ardakani

**Affiliations:** 1 Department of Biomedical and Molecular Sciences, Queen’s University, Kingston, ON K7L 3N6, Canada; 2 Scientific Research Division, Novateur Ventures Inc., Vancouver, BC V6E 3P3, Canada; craiglaferriere@gmail.com (C.L.); ali@novateur.org (A.A.)

**Keywords:** COVID-19, SARS-CoV-2, vaccine, target product profile, immune response, coronavirus, clinical trial, public health

## Abstract

The coronavirus SARS-CoV-2, which causes Coronavirus disease 2019 (COVID-19), has infected more than 100 million people globally and caused over 2.5 million deaths in just over one year since its discovery in Wuhan, China in December 2019. The pandemic has evoked widespread collateral damage to societies and economies, and has destabilized mental health and well-being. Early in 2020, unprecedented efforts went into the development of vaccines that generate effective antibodies to the SARS-CoV-2 virus. Teams developing twelve candidate vaccines, based on four platforms (messenger RNA, non-replicating viral vector, protein/virus-like particle, and inactivated virus) had initiated or announced the Phase III clinical trial stage by early November 2020, with several having received emergency use authorization in less than a year. Vaccine rollout has proceeded around the globe. Previously, we and others had proposed a target product profile (TPP) for ideal/optimal and acceptable/minimal COVID-19 vaccines. How well do these candidate vaccines stack up to a harmonized TPP? Here, we perform a comparative analysis in several categories of these candidate vaccines based on the latest available trial data and highlight the early successes as well as the hurdles and barriers yet to be overcome for ending the global COVID-19 pandemic.

## 1. Introduction

The pandemic of 2020 caused by the global dissemination of the SARS-CoV-2 coronavirus has crippled the world. Tens of millions of infections and over two million deaths (https://coronavirus.jhu.edu/map.html (accessed on 9 February 2021)) from coronavirus disease 2019 (COVID-19) have given rise to sorrow, social isolation, and disruption of life as we know it. An immense body of the literature has been accumulating in the past year explaining the epidemiology and infectious mechanisms of SARS-CoV-2, the pathogenesis of COVID-19, and the development of potential therapies and vaccines [1,2,3,4,5,6,7,8,9,10,11,12,13,14]. In June 2020, we had previously provided a global snapshot of the race for an approved vaccine targeting SARS-CoV-2 and outlined what the ideal vaccine should look like via a proposed target product profile (TPP) [15]. A TPP for a COVID-19 vaccine defines a preferred and minimally acceptable outline for generation of immunity to protect populations from the risk of disease and for reactive use in pandemic outbreaks. Currently, there are more than 200 vaccines at various stages of development specifically targeting SARS-CoV-2 according to the latest World Health Organization (WHO) database (https://www.who.int/publications/m/item/draft-landscape-of-covid-19-candidate-vaccines (accessed on 9 February 2021)). Of these, twelve had reached/announced Phase III clinical trials by mid-November 2020, having initiated development months before this time. Several more have attained this stage in the ensuing months but we are limiting our analysis to these first twelve candidates. A comparative chart of guidelines for a COVID-19 vaccine TPP from four sources (ours [15], WHO (https://www.who.int/publications/m/item/who-target-product-profiles-for-covid-19-vaccines (accessed on 9 February 2021)), Coalition for Epidemic Preparedness Innovations (CEPI) (https://media.tghn.org/articles/TPC_COVID-19_CEPI_Version_1.1_20200303.pdf (accessed on 9 February 2021)) and Center for Biologics Evaluations and Research (CBER) (https://www.fda.gov/media/139638/download (accessed on 9 February 2021)) is presented in Table S1 of the Supplementary Materials.

Gavi, the Vaccine Alliance, with its associated COVAX vaccine pillar of Access to COVID-19 Tools (ACT) Accelerator (https://www.gavi.org/ (accessed on 9 February 2021); https://www.gavi.org/covax-facility (accessed on 9 February 2021)), which includes CEPI and WHO partnerships, in addition to Operation Warp Speed (to be renamed) (https://www.bloomberg.com/news/articles/2021-01-15/biden-team-is-retiring-warp-speed-name-from-vaccination-push (accessed on 9 February 2021)) and other company initiatives have paved the way for the rollout of vaccine candidates to the world’s population. This includes safety testing, manufacturing, distribution and logistics for providing billions of doses to combat COVID-19 and end the global pandemic. Here, we update and revise the outlook of the most advanced candidate vaccines in five (of fourteen; see Table S1) categories (safety/reactogenicity, efficacy/immunogenicity, dosing regimen, product stability/storage/supply chain (i.e., logistics) and target price/accessibility) to see how they stack up to a harmonized TPP (see below).

## 2. Overview of Twelve Vaccines Targeting COVID-19 in Phase III Clinical Trials

**Figure 1:**
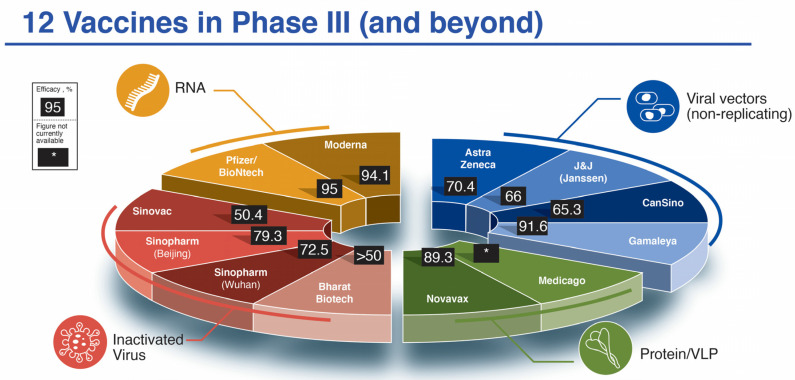
The twelve SARS-CoV-2 vaccine candidates reaching/announcing Phase III clinical trials by mid-November 2020. Shown in pie-chart configuration are the companies responsible for the development of the vaccines as well as their reported efficacy in Phase III trials. *, efficacy not yet available. Due to variability in reporting criteria for cases of COVID-19, efficacy results may not be directly comparable.

Within ten months’ time, twelve vaccine candidates had been officially registered into Phase III clinical trials (https://www.who.int/publications/m/item/draft-landscape-of-covid-19-candidate-vaccines (accessed on 9 February 2021)). This is a phenomenal and unprecedented feat in the history of medicine, owing to a worldwide concerted effort to eradicate this viral pandemic. The process of vaccine development normally takes place over several years but has now been accelerated through concurrent pre-clinical and early Phase I studies, strategic risk measures and adaptive trial designs. Here, we consider vaccine candidates, two each from the messenger RNA and protein/virus-like protein platforms and four each from the viral vector (non-replicating) and inactivated virus platforms (Figure 1). Each of these platforms, and 9 of 12 vaccine candidates were covered partially in our previous publication [15]. We will briefly review and update the salient characteristics of each here (Table 1).

**Table 1:** Summary of Vaccines Analyzed in this Study.

Platform [Ref.]	Name (Dose)	Phase III # Enrolled Placebo	Phase III # Enrolled Vaccine	COVID Cases Placebo (Severe)	COVID Cases Vaccine (Severe)	% Efficacy (Severe)	Projected Cost/Dose (USD)
**RNA**							
Pfizer/BioNTech [16]	BNT162b2 (2 × 30 μg, 21 days apart)	21,720	21,728	162 (9)	8 (1)	95 (89)	$14.70–19.50
Moderna [17]	mRNA-1273 (2 × 100 μg, 28 days apart)	15,210	15,210	185 (30)	11 (0)	94.1 (100)	$25–37
**Viral Vector**							
Astra Zeneca ^a^ [18]	AZD1222 (2 × 5×10^10^ vp, 28 days apart) (2.2 × 10^10^ vp, then 5 × 10^10^ 28 days apart)	4455 [1374]	4440 [1367]	71 [30] (10/2)	27 [3] (0/0)	62.1 (70.4) [90] (100)	$2–10^+^
CanSino ^b^	Ad5-nCoV (Convidecia) (single dose 5 × 10^10^ vp)	30,000 (Pakistan)		---	---	65.37 (91)	$ < 4
Gamaleya ^c^ [19]	Sputnik V (Gam-COVID-Vac) (10^11^ vp rAd26-S, followed by 10^11^ vp rAd5-S 21 days apart)	5476	16,501	62	16	91.6	$ < 10
J&J (Janssen) ^d^	Ad26.COV2.S (single dose 5 × 10^10^ vp)	19,178	19,306	193 (34)	66 (5)	66 (85.4)	$2.80–9
**Protein/VLP**							
Novavax ^e^	NVX-CoV2373 (2 × 5 μg + 50 μg M1 adjuvant, 21 days apart)	15,000^+^ (UK)		56 (1)	6 (0)	89.3 (UK)	$16
Medicago ^f^	CoVLP (2 × 3.75 μg + AS03 GSK adjuvant, 21 days apart)	30,000		----	---	---	Not known
**Inactivated**							
Sinovac ^g^	CoronaVac (2× alum-adjuvanted 14 days apart)	9200 (Brazil)		167 (31)	85 (7)	50.4 (Brazil)	$30
Sinopharm ^h^ (Beijing)	BBIBP-CorV (2 × 4 μg in alum adjuvant 21 days apart)	10,000 (UAE)		----	---	79.3 (UAE)	$30–72.50
Sinopharm ^i^ (Wuhan)	Vero (2 × 5 μg in alum adjuvant 21 days apart)	27,000 (Peru, UAE, Bahrain, Morocco, Argentina, Jordan and Pakistan)		---	---	72.5%	$30–72.50
Bharat Biotech ^j^	COVAXIN (BBV152) (2 × 6 μg in Alhydroxiquim-II adjuvant 28 days apart)	25,800		---	---	>50%	$2–3

Notes: Numbers assigned to vaccine and placebo groups are always greater than numbers used in the per protocol analysis due to patient dropout and other variables. Numbers enrolled, in some cases, include the published interim analyses, so these may be far below actual participants in the Phase III trials. Some are based on projections. For each vaccine candidate, there are different criteria for defining mild, moderate and severe cases, as well as overall efficacy. Projected costs have appeared from multiple sources and can vary substantially depending on negotiated agreements. Here are a few examples: https://www.bbc.com/news/world-asia-china-55212787, https://www.globaltimes.cn/content/1210093.shtml and https://gulfnews.com/world/revealed-prices-of-some-leading-covid-19-vaccine-candidates-1.1598192772387?slide=10. ^a^ For AZ/Ox, the placebo group had 10 hospitalized/2 severe (1 death) and for AZD1222 0 hospitalized/0 severe. Efficacy is presented separately for the two different dosing regimens (incorrect one in square parentheses) and combined (in round parentheses). More recent analyses [20] indicated that vaccine efficacy could be increased to 82.4% for AZD1222 in the UK when the dosing interval was increased to 12+ weeks. These updated data are not included since they deviate from the original trial designation. ^b^ There has been a dearth of information on this vaccine’s results in advanced clinical trials. Preliminary results from a study in Pakistan (https://www.bloomberg.com/news/articles/2021-02-08/pakistan-says-cansino-s-covid-vaccine-shows-65-7-efficacy (accessed on 9 February 2021)) were released on 8 February 2021 but number of COVID cases and breakdown of cases between placebo and vaccine were lacking. Apparently, the vaccine shows 91% efficacy for severe disease. ^c^ More details about the vaccine can be found here: https://sputnikvaccine.com/about-vaccine/. ^d^ Data from theVRBPAC document [21]. Efficacy showed regional variation, highest in the USA (72%), followed by Latin America (66%), then South Africa (57%). Presumably, the 95% prevalence of the B.1.3.351 variant in Africa reduced efficacy. Protection against severe disease was 85% across all regions and was 100% when considering hospitalization and death. ^e^ Data from trial data released on 28 January 2021 at: https://ir.novavax.com/news-releases/news-release-details/novavax-covid-19-vaccine-demonstrates-893-efficacy-uk-phase-3. Efficacy calculated against the “original” SARS-CoV-2 strain was 95.6% and 85.6% against the UK variant B.1.1.7 strain. In a South Africa Phase 2b clinical trial of 4400 participants, 60% efficacy for the prevention of mild, moderate and severe COVID-19 disease was observed in 94% of the study population that was HIV-negative with 44 total cases (29 placebo, 15 in the vaccine group). One severe case occurred in the placebo group and all other cases were mild or moderate. Preliminary indications show that 25 of the 44 COVID-19 cases) were the South Africa B.1.3.351 variant. ^f^ After initially announcing the start of Phase II/III trials in early November 2020, the Phase III component has yet to start as of February 10, 2021. ^g^ There has been considerable variability in reporting efficacy data for this vaccine candidate. Data are presented from the most complete dataset from trials in Brazil at https://www.bbc.com/news/world-latin-america-55642648. The vaccine trial data from Brazil are apparently 78% effective in preventing mild cases and 100% effective in staving off moderate to serious cases. In trials in Turkey, the vaccine was 91.25% effective, while in Indonesia, it was 65.3% effective. These interim results need to be amalgamated and thoroughly examined. ^h^ Data are scant on this vaccine with two figures appearing on interim vaccine data efficacy at: https://www.reuters.com/article/health-coronavirus-vaccine-china-idUSKBN297035. Earlier data had indicated an 86% efficacy in UAE (https://www.reuters.com/article/health-coronavirus-china-vaccine-int-idUSKBN2940CA). ^i^ Data are scant on this vaccine. https://www.dw.com/en/coronavirus-how-effective-are-the-chinese-vaccines/a-56370802 and https://www.reuters.com/article/us-health-coronavirus-vaccine-sinopharm-idUSKBN2AO0WW. ^j^ Data from Phase III have not been revealed as of 10 February 2021 and it is only assumed the minimum efficacy designated by the WHO will be achieved based on its EUA in India (see https://theprint.in/health/even-if-covaxin-doesnt-meet-efficacy-mark-govt-has-no-plan-to-re-vaccinate-those-taking-it/589910/ and https://indianexpress.com/article/explained/coronavirus-vaccines-india-covishield-bharat-biotech-covaxin-7131057/).

### 2.1. Messenger RNA (mRNA) Vaccines

The Moderna mRNA-1273 [22] and Pfizer/BioNtech BNT162b2 (also known by brand name Comirnaty or by generic name tozinameran) (https://www.fiercepharma.com/marketing/pfizer-biontech-select-comirnaty-as-brand-name-for-covid-19-vaccine (accessed on 9 February 2021)) [23] mRNA vaccines have been the frontrunners in the vaccine “race” since early 2020 when the pandemic was first declared [15]. Although an mRNA vaccine has never before been approved by regulatory agencies, this has now changed with the United States Food and Drug Administration (FDA) emergency use authorization (EUA) of these two candidates [24] in December 2020, after an astoundingly short 11 month journey, a process that normally takes place over 10+ years. Both vaccines employ an mRNA, which targets the pre-fusion stabilized SARS-CoV-2 full-length spike P2-mutant version, as the immunogen, wrapped in proprietary lipid nanoparticle (LNP) formulations (see [24] and Supplementary Materials Table S2 for the precise compositions of the LNPs) in a prime-boost format (Pfizer/BioNTech: 30 μg immunogen, 21 days apart and Moderna 100 μg immunogen, 28 days apart). Both have documented 94–95% efficacy based on 30-44,000 participants in their respective Phase III double-blinded, randomized placebo-controlled studies ([16,17]; see Figure 1, Table 1). These remarkably positive findings overwhelmingly exceeded early expectations.

### 2.2. Viral Vector-Based (Non-Replicating) Vaccines

Four groups (Astra Zeneca/University of Oxford (AZ/Ox), CanSino Biologics, Gamaleya Research Institute and Johnson & Johnson/Janssen (J&J)) have all adopted the use of non-replicating adenoviral vectors to drive the expression of the full-length SARS-CoV-2 spike glycoprotein (5 × 10^10^ or 10^11^ viral particles for each dose injection) to induce an immune response [18,19,25,26,27,28,29,30,31]. The differences between them lie in the chosen vector strain and immunization schedules. All except J&J use a prime-boost scheme. AZ/Ox (vaccine named AZ1222 or Covishield in India) (https://www.bbc.com/news/world-asia-india-55748124 (accessed on 9 February 2021)) uses an adenoviral vector that infects chimpanzees but not humans [25,26]. CanSino (Ad5-nCoV) uses a recombinant version of Ad5 adenovirus that naturally transmits in humans [28,29], while J&J (Ad26.COV2.S) uses a variant adenovirus Ad26 that is not normally (or only rarely) encountered by the human population [31]. Gamaleya (Sputnik V) uses a combination prime with Ad26 and boost with Ad5 [19,30]. From 12–40,000^+^ have been enrolled in Phase III trials for each of the four members of this platform. Efficacy results have ranged from promising (91.6% for Sputnik V) to variable (62–90% for AZ/Ox where a clinical trial “mistake” led to a surprising increased efficacy) [18,32,33] (see Figure 1, Table 1).

### 2.3. Recombinant Protein-Based Vaccines

The protein subunit Novavax candidate NVX-CoV2373 uses the recombinant, properly folded, full-length SARS-CoV-2 spike glycoprotein in the pre-fusion state engineered from insect cells in a nanoparticle formulation along with their proprietary saponin-based Matrix-M adjuvant [34]. Medicago also utilizes the full-length spike glycoprotein with several key changes: (i) a plant gene signal peptide; (ii) three stabilizing substitutions at the S1/S2 cleavage site; (iii) the dual proline (P2) prefusion conformation-stabilizing exchanges; and (iv) transmembrane and cytoplasmic tail sections swapped with influenza sequences) expressed in tobacco (*Nicotiana benthamiana*) plants [35]. Swapping the influenza domains increases viral-like protein (VLP) assembly and budding. Medicago utilizes the AS03 adjuvant, an oil-in-water emulsion containing tocopherol and squalene supplied by GlaxoSmithKline, which is mixed immediately prior to administration. Both use a prime-boost regimen 21 days apart with either 5 (Novavax) or 3.75 (Medicago) μg protein/dose. Early indications show excellent efficacy (just below 90%) (https://ir.novavax.com/news-releases/news-release-details/novavax-covid-19-vaccine-demonstrates-893-efficacy-uk-phase-3 (accessed on 9 February 2021)) for NVX-CoV2373.

### 2.4. Inactivated Virus

Three Chinese conglomerates and one Indian company have used the tried-and-true method of viral inactivation by β-propriolactone treatment, along with various alum adjuvants for generation of their SARS-CoV-2 vaccine candidates [36,37,38,39,40,41,42]. Each is using the prime-boost regimen with 4–6 μg virus and 14–28 days separation between prime and booster injections.

## 3. Clinical Trial Data of Twelve Vaccines Targeting COVID-19 in Phase III Trials and Emergency Use Authorization

### 3.1. mRNA Vaccines—Safety/Reactogenicity and Immunogenicity/Efficacy

The Phase III data for both Pfizer/BioNTech and Moderna vaccines that are built on the same platform, with nearly identical antigen-producing mRNA sequences and their own unique blend of LNP encapsulation, yielded remarkably similar results in terms of safety and efficacy [16,17,22,43,44]. The most common solicited local and systemic reactogenicity were pain at the injection site, headache, and fever (defined as temperature ≥ 38 °C). In most cases, these adverse events (AEs) were reported for 7 days post-injection (Table 2). In general, AEs were more abundant after the boosting dose. Table 2 shows that mRNA vaccines induced fever in 0.8–3% of subjects after the first dose. The mRNA vaccines have a reactogenicity profile similar to other vaccines commonly used in adults. For example, the pneumonia vaccine Prevnar^®^ 13 induced fever in the range 1.0–7.2% of subjects (Prevnar Product Monograph [45]). The shingles vaccine Shingrix induced fever (>37.5 °C) in 14.3–25.9% of adult subjects aged 50 years and above (Shingrix Product Monograph [46]). The HPV vaccine Gardasil^®^ induced fever (>37.8 °C) in 2.5% of women aged 27 to 45 years (Gardasil-9 Product Monograph [47]). Influenza vaccine tends to induce less fever, around 1–2% for adults over 18 years of age (Fluviral product monograph [48]).

**Table 2:** Solicited reactogenicity 7 days post injection, all ages, Phase 3 or Phase 1/2 data of the chosen final formulation and schedule.

	Dose	N	Injection Site Pain		Headache		Fever	
Grade			Any		Any		Oral ≥ 38 °C	
Group		Placebo + Vaccine	Placebo	Vaccine	Placebo	Vaccine	Placebo	Vaccine
**mRNA**								
Pfizer/BioNTech BNT162b2 (2 × 30 μg, 21 d.a.) [16,43]	Post dose 1	~8183 ^a^	12%	78%	27%	35%	0.6%	3%
	Post dose 2	~8183 ^a^	10%	73%	20%	47%	0%	14%
Moderna mRNA-1273 (2 × 100 μg, 28 d.a.) [17,22,44]	Post dose 1	~30,300 ^b^	17.5%	83.7%	26.6%	32.7%	0.3%	0.8%
	Post dose 2	~29,200 ^b^	17.0%	88.2%	23.4%	58.6%	0.3%	15.5%
**Viral Vector**								
Astra Zeneca AZD1222 (2 × 5 × 10^10^ vp, 28 d.a.) [18,25,26]	Post dose 1	964 ^c^	37%	67%	41%	68%	2%	18%
	Post dose 2	128 ^d^	---	30.7%	---	%	---	0%
CanSino Ad5-nCoV (single dose 5 × 10^10^ vp) [28,29]	Post dose 1	255 ^e^	9%	56%	13%	28%	10% ^f^	16% ^f^
	Post dose 2	NA	NA	NA	NA	NA	NA	NA
Gamaleya Sputnik V (10^11^ vp Ad26 then 10^11^ Ad5 21 d.a.) [30]	Post dose 1	9 ^g^	---	78%	---	67%	---	89% ^h^
	Post dose 2	20 ^g^	---	40%	---	55%	---	100% ^h^
J&J (Janssen) (Ad26.COV2.S single 5 × 10^10^ vp) [21,49]	Post dose 1	673 ^j^	16.7%	48.7%	23.9%	39.2%	0.9%	9.1%
	Post dose 2	NA	NA	NA	NA	NA	NA	NA
**Protein/VLP**								
Novavax NVX-CoV2373 (2 × 5 μg+ 50 μg M1, 21 d.a.) [34]	Post dose 1	49 ^k^	13%	38%	31%	23%	0%	0%
	Post dose 2	49 ^k^	10%	58%	29%	47%	0%	0%
Medicago CoVLP 2 × 3.75 μg+ AS03, 21 d.a. [35]	Post dose 1	20 ^m^	---	95%	---	30%	---	0%
	Post dose 2	19 ^m^	---	100%	---	57.9%	---	36.8%
**Inactivated**								
Sinovac CoronaVac (2 × 3 µg + alum 14 d.a.) [36,37]	Post dose 1	180 ^p^	10%	9.2%	1.7%	0.8%	1.7%	2.5%
	Post dose 2	180 ^p^	3.3%	13.6%	0%	0.8%	0%	1.7%
Sinopharm BBIBP-CorV (2 × 4 μg vp + alum, 21 d.a.) [38]	Post dose 1 or 2 ^q^	112	4%	12%	7%	1%	4%	4%
	Post dose 2	---	---	---	---	---	---	---
Sinopharm (Wuhan) Unnamed (2 × 5 μg vp + alum, 21 d.a.) [39]	Post dose 1	112 ^r^	14.3%	6.0%	0%	0%	3.6%	1.2%
	Post dose 2	112 ^r^	10.7%	9.5%	0%	0%	0%	1.2%
Bharat Biotech COVAXIN (2 × 6 μg Alhydroxiquim-II 28 d.a.) [42]	Post dose 1	175 ^s^	---	3.2%	---	0.5%	---	4.2%
	Post dose 2	175 ^s^	---	2.6%	---	1.5%	---	2.1%

Notes: The most common solicited local and systemic reactogenicity were pain at the injection site, and headache, and these have been reported in Table 2 for 7 days following injection, except where noted. Reported also is fever defined as temperature ≥ 38 °C by oral route, except where noted. ^a^ 16–55 years and >55 years combined. ^b^ VRBPAC document, Tables 21 and 22. November 25 dataset. ^c^ Ages 18–55 y.o. No paracetamol. See Table S2 from reference [25]. ^d^ Ages 18+ y.o., mean of 3 age groups combined. See Table S11 from reference [27].^e^ See Table 2 from reference [28]. ^f^ The definition of fever was not provided in the publications. Severe fever was defined > 38.5 °C [29]. ^g^ Data reported for the liquid formulation using Ad26 +Ad5 schedule from Table 2 in reference [30]. ^h^ Fever defined as ≥ 37.0 °C, body location not indicated. ^j^ Ages ≥ 18 to ≤60 years and ≥60 years combined. Extracted from Figures 22 and 23 in reference [21]. ^k^ Ages 18–59 years. Data extracted from Figure 2 in reference [34]. ^m^ SARS-CoV-2 seronegative adults (18–55 years). Supplementary Table S4 in reference [35]. ^p^ Adults aged 18–59 years. Reported within 14 days post first dose and 28 days post dose 2. Phase 2 data from Tables A3–A6 in Appendix 2 in reference [36]. ^q^ Adults aged 18–59 years. Fever not defined. Data are 7 days after 1st or/and 2nd doses combined. Table 5 in reference [38]. ^r^ Adults aged 18–59 years. Fever defined as axillary temperature >37.0 °C. Phase 2 data from Supplement 3 eTable 2 in reference [39]. ^s^ Children and adults aged 12–64 years. Phase 2 data. Table 3 in reference [42].

Efficacy for both mRNA vaccine candidates was nearly identical at 94–95% [16,17]. These results represent a resounding success for the novel platform technology, which has never previously received approval for any vaccine or drug program. Since receiving emergency use authorization (EUA) approval, severe allergic reaction cases have arisen during vaccine rollout [50,51], which required emergency epinephrine rescue [52]. The case numbers exceed the typical rare “1 in a million” side effects that emerge in the general population after clinical trials, where exclusion criteria limit involvement of allergic individuals and other potentially vulnerable participants. Early speculation centers around an adverse anaphylactoid reaction to the polyethylene glycol (PEG) that is found in both formulations [52,53]. The mechanism of action is not understood, but hypotheses include IgE antibodies to PEG, non-IgE complement activation-related pseudo-allergy (CARPA), nanoparticle aggregation and a negatively charged linkage of lipid to PEG [52,53]. There appears to be a statistically significant difference in the rate of allergic reactions between the Pfizer and Moderna vaccines (~11.1 [50] vs. ~2.5 [51] per million injections, respectively; *p* < 0.001, Fisher’s exact) and a comparison of the Pfizer and Moderna lipid nanoparticle formulations presented in the Supplementary material Table S2 shows that the difference in the lipidated-PEG molecules between these two vaccines is at the linkage between the lipid and the PEG. The two vaccines also have different cationic lipids, SM-102 and ALC-0315, but these have not been implicated in the allergic reactions.

Table 3 shows the immunogenicity data. A clear booster effect was observed after the second dose of both mRNA vaccines in both total anti-SARS-CoV-2 IgG and neutralization titers. There also appeared to be an increase in the proportion of spike-specific interferon (IFN)-γ CD4^+^ T cells after booster with the Moderna vaccine, but data are lacking for the post-first dose Pfizer vaccine at this time. Both mRNA vaccines showed evidence of a T helper (Th)1 bias.

**Table 3:** Immune response as measured by IgG, virus neutralization, proportion of γ-IFN^+^ CD4^+^ T cells and Th1/Th2 ratio of CD4^+^ T-cells, post vaccination, all ages, from Phase 3 or Phase 1/2 using the final chosen formulation and schedule.

	Timing	N	ELISA Titer		Virus Neutralization		γ-IFN^+^ CD4^+^ T Cells	Th1/Th2 CD4^+^ T cells
Units	dpd = Days Post Dose		GMT IgG		GMT = Geometric Mean Titer		%^+^	Ratio of Measure
Group		P + V	Placebo	Vaccine	Placebo	Vaccine	Vaccine	Vaccine
**mRNA**								
Pfizer/BioNTech BNT162b2 (2 × 30 μg, 21 d.a.) [16,43]	21 dpd 1	~30 ^a^	0.8	665.7 ^c^	10.0	13.0 ^d^	---	---
	31 dpd 2	360 ^b^	0.8	4931.7 ^a,c^	10.0	316.1 ^d^	0.08% ^e^	4.4 ^f^
Moderna mRNA-1273 (2 × 100 μg, 28 d.a.) [22,44]	29 dpd 1	~180 ^g^	5.80	25.23	21.0	149.3 ^h^	0.02% ^j^	>>1 ^j^
	28 dpd 2	~180 ^g^	5.86	147.42	21.2	1095.8 ^h^	0.09% ^j^	28.5 ^j^
**Viral Vector**								
Astra Zeneca AZD1222 (2 × 5 × 10^10^ vp, 28 d.a.) [18,25,26,27]	28 dpd 1	127	1 ^k^	546 ^k^	23.9 ^m,^	203.2 ^m^	0.06% ^p^	6.3 ^q^
	28 dpd 2	112	---	10,691 ^n^	---	128 ^n^	0.097% ^n^	---
CanSino Ad5-nCoV (single dose 5 × 10^10^ vp) [28,29]	28 dpd 1	255	20 ^r^	571 ^r^	4 ^s^	18.3 ^s^	0.01% ^t^	---
	NA	NA	NA	NA	NA	NA	NA	NA
Gamaleya Gam-COVID-Vac (1 × 10^11^ vp Ad26 then Ad5 21 d.a.^a^) [19,30]	21 dpd 1	9	12.5 ^u^	1721 ^u^	1.25 ^v,w^	4.2 ^v^	--- ^x^	--- ^x^
	21 dpd 2	456	---	8996 ^u^	1.6 ^v^	44.5 ^v^	---	---
J&J (Janssen) (Ad26.COV2.S single dose 5 × 10^10^ vp) [21,49]	29 dpd 1	~314	<50	399 ^y^	<58	241 ^z^	0.09% ^α^	10 ^β^
	NA	NA	NA	NA	NA	NA	NA	NA
**Protein/VLP**								
Novavax NVX-CoV2373 (2 × 5 μg + 50 μg M1, 21 d.a.) [34]	21 dpd 1	~52	110 ^δ^	1984 ^δ^	20 ^ε^	103 ^ε^	---	---
	14 dpd 2	~50	114 ^δ^	63,160 ^δ^	20 ^ε^	3906 ^ε^	0.18% ^ζ^	7.5 ^ζ^
Medicago CoVLP (2 × 3.75 μg + AS03, 21 d.a.) [35]	21 dpd 1	20	64.8 ^w,η^	4354 ^η^	5 ^w,θ^	29.3 ^θ^	0.01% ^λ^	2.37 ^λ^
	21 dpd 2	19	---	295,240 ^η^	---	811.3 ^θ^	0.06% ^λ^	1.4 ^λ^
**Inactivated**								
Sinovac CoronaVac (2 × 3 µg + alum, 14 d.a.) [36,37]	14 dpd 2	176 ^ξ^	81	1094.3	2.0	27.6	0.00% ^π^	---
	28 dpd 2	176 ^ξ^	80	1053.7	2.0	23.8	0.07% ^π^	---
Sinopharm (Beijing) BBIBP-CorV 2 × 4 μg vp + alum, 21 d.a. [38]	14 dpd 1 ^ς^	112	---	---	2	219	---	---
	28 dpd 2 ^ς^	112	---	---	2	282	---	---
Sinopharm (Wuhan) Unnamed 2 × 5 μg vp + alum, 21 d.a. [39]	14 dpd 1 ^σ^	56	---	---	---	---	---	0.92 ^φ^
	14 dpd 2 ^σ^	56	10	215	5	247		1.21 ^φ^
Bharat Biotech COVAXIN (2 × 6 μg in Alhydroxiquim-II adjuvant, 28 d.a.) [40,41,42]	28 dpd 1 ^ψ^	190	500 ^w^	2240	5.6 ^w^	12.8	---	---
	28 dpd 2 ^ψ^	190	---	9541	---	160	---	42 ^ω^

^a^ Study C4591001 Phase 1. Weighted average for all ages combined. ^b^ Study C4591001 Phase 2. Weighted average for all ages combined. ^c^ SARS-CoV-2 S1 binding IgG direct Luminex immunoassay. ^d^ Neutralization assay used SARS-CoV-2 (USA_WA1/2020) that had been rescued by reverse genetics and engineered by the insertion of mNeonGreen (mNG) described in reference [54]. ^e^ Study BNT162-01 Phase 1. N = 10 subjects. See Figure 2 in reference [43]. ^f^ Study BNT162-01 Phase 1. Cytokine-producing T cells were identified by intracellular cytokine staining. See Figure 3 in reference [43]. Samples drawn 7 days post boost, 10 subjects, INFγ/IL4 ratio. ^g^ Study 201. Tables 14 and 15 in reference [44]. ^h^ Measured by a microneutralization (MN) assay; details not provided. ^j^ 28 dpi 1 and 14 dpi2 extracted from Figure S9B in reference [22]. N ≈ 15. Ratio = Any Th1/any Th2 Peptide pool S1. No observed Th2 response post injection 1. ^k^ IgG response by standardized ELISA to spike protein in trial participants. N = 127. Extracted from Figure 3 in reference [25]. ^m^ Public Health England microneutralization (strain not mentioned) IC_50_ median values extracted from Figure 3 in reference [25]. Ages 18-55 y.o. N = 45 for 1st dose. ^n^ Ages 18+ y, weighted mean for all ages, N = 112. CMI 14 days post-dose 2, N = 101, from reference [27]. ^p^ Interferon-γ ELISpot response to peptides spanning the SARS-CoV-2 spike. Median values per PBMC. Data extracted from Figure 6 in reference [25]. ^q^ Ratio of any Th1 to any Th2 cytokine secretion by CD4^+^ T cells at day 14 after vaccination (n = 34). From Figure 4e in reference [26]. ^r^ ELISA antibodies to receptor binding domain using ELISA kits (Beijing Wantai BioPharm, Beijing, China). Values extracted from Figure 2B in reference [28]. ^s^ Neutralizing antibodies to live SARS-CoV-2, strain human/CHN/Wuhan_IME-BJ01/2020, GenBank number MT291831.1. Values extracted from Figure 2C in reference [28]. ^t^ The cellular immune responses of the expression of interferon (IFN) γ stimulated by the overlapping peptide pool of spike glycoprotein were detected by enzyme-linked immunospot (ELISpot) assay (Mabtech, Stockholm, Sweden). Values per 100,000 PBMC were extracted from Figure 3A in reference [28]. ^u^ Receptor binding domain specific antibodies as measured by ELISA extracted from Figure 2A in reference [30]. Glycoprotein S1 ELISA titer (not shown here) are available in Figure S1 from reference supplemental material. ^v^ N = 100. GMT measured by microneutralization assay using SARS-CoV-2 (hCoV-19/Russia/Moscow_PMVL-1/2020) with 50% tissue culture infective dose (TCID_50_) of 100. Data extracted from Figures 2C and 2D in reference [30] and from reference [19]. ^w^ Pre-immune sera. ^x^ T cell Interferon-γ responses were reported in reference [30], but the units do not correspond with the other studies summarized here. A request for clarification was not answered by the author. ^y^ Brazil, South Africa and USA combined. SARS-CoV-2 Spike S binding antibodies in serum as measured by ELISA extracted from Figure 7 in reference [21]. ^z^ Ages 18-55 and 65^+^ y.o. combined. SARS-CoV-2 serum neutralizing antibody titers were measured in a microneutralization wtVNA using the Victoria/1/2020 SARS-CoV-2 136 strain at Public Health England (PHE). Data from Figure 3 in reference [21]. ^α^ Ages 18-55 and 65^+^ y.o. combined. Median % CD4^+^ cells expressing IFN-γ and/or IL-2 after stimulation with SARS-CoV-2 S protein peptide pool in peripheral blood mononuclear cells 29 days post vaccination. Extracted from Figure 5 in reference [21]. ^β^ Ratio of average median values of Th1/Th2 from 5 × 10^10^ vp Cohort 1a and Cohort 3 from Figure 2C in reference [49]. ^δ^ SARS-CoV-2 Spike Protein Serum IgG ELISA performed at Novavax Clinical Immune Laboratory (Gaithersburg, MD). GMT from Table S10 in reference [34]. ^ε^ SARS-CoV-2 Microneutralization Assay (using strain USA-WA1/2020 BEI Resources NR-52281) performed at the University of Maryland School of Medicine, Baltimore, MD. GMT from Table S11 in reference [34]. ^ζ^ N = 4, 7 days post dose 2. Average % of IFN-γ producing T cells extracted from Figure 5C in reference [34]. Ratio of average of all 3 Th1 to both Th2. ^η^ CoVLP-induced Binding Antibody IgG ELISA using SARS-Cov2 Spike protein (SARS-Cov2/Wuhan/2019, Immune Technology Corp. GMT from Supplementary Table 10 in reference [35]. ^θ^ Live Wild-Type SARS-CoV-2 (strain 2019 nCOV ITALY/INMI1, provided by EVAg; Genebank: MT066156) microneutralization cytopathic effect-based assay (VisMederi, Sienna, Italy). GMT from Supplementary Table S12 in reference [35]. ^λ^ CoVLP-induced IFNγ Cellular Response (ELISpot) was performed using an IFN-γ ELISpot assay (Human IFN-γ ELISpot assay, Cellular Technology Limited (CTL), USA). Median % Spot Forming Units from Supplementary Table 13 in reference [35]. Induced IL-4 Cellular Response (ELISpot) was performed using an IL-4 ELISpot assay (Human IL-4 ELISpot assay, Cellular Technology Limited (CTL), Cleveland, OH, USA). The ratio of Th1/Th2 was calculated by the IFNγ/IL-4 median % Spot Forming Units. ^ξ^ Phase 2 data from Table 3C and 3G in reference [36]. RBD-specific IgG were quantified using the in-house ELISA kit from Sinovac. Neutralizing antibodies to live SARS-CoV-2 (virus strain SARS-CoV-2/human/CHN/CN1/2020, GenBank number MT407649.1) were quantified using a micro cytopathogenic effect assay. ^π^. ELISpot Method adopted with S protein overlapping peptide T cell responses. N = 24, 14 days post dose 1 and 14 days post dose 2, mean Phase 1 data from Table A7-1 in Appendix 7 in reference [36]. ^ς^ Phase 2 data. Assay using infectious SARS-CoV-2(strain not identified) neutralizing assay and expressed as GMT. Data from Appendices 17 & 18 in reference [38]. Data are 28 dpd 1 or 28 dpd 2. ^σ^ Phase 2 data. ELISA against purified whole-virus. Neutralization performed using Plaque Reduction Neutralization Test, virus strain not specified. Table 3 in reference [39]. ^φ^ Ratio of Th1 vs. Th2 cytokine concentration (IFN-gamma divided by IL-4) from Changes in T Helper 1 Cell Related Cytokines in the Phase 1 Trial. Data extracted from Supplement 3 eFigure 3 and eFigure 4 from reference [39]. ^ψ^ Anti-IgG responses against the spike (S1) protein GMT from Table 2. Microneutralization assay (MNT50) GMT. Details from the supplementary material not available at the time of writing. Extracted from Figure 2C in reference [42]. ^ω^ Ratio of cytokine levels in supernatants after stimulation with SARS-CoV-2 peptides, N = 29, 2 weeks post dose 2. Th1/Th2 cytokines including IFNγ, TNF-α, IL-2/IL-5, IL-10, IL-13. Extracted from Figure 4 in reference [42].

### 3.2. Viral Vector-Based (Non-Replicating) Vaccines—Safety/Reactogenicity and Immunogenicity/Efficacy

AZ/Ox was first to release their published, peer-reviewed Phase III clinical trial data on 8 December 2020 based on an interim analysis of data out of sites in the UK, South Africa and Brazil [18]. Overall efficacy data based on 11,636 participants indicated a vaccine efficacy of 70.4%. However, the waters were muddied somewhat when it was revealed that results were broken down into two different regimens. One subgroup of 2738 subjects received only half of the initial priming dose resulting in a 90% efficacy rate, while the larger group received the two standard doses of viral vector vaccine (Table 1), which resulted in a 62.1% efficacy rate. As for safety, in general, severe adverse events (SAEs) were distributed equally among placebo and vaccine groups, in contrast to the earlier safety trials [25], which indicated greater reactogenicity in the vaccine relative to the placebo group in terms of fever, pain, and headaches (Table 2). The viral vector vaccines presented in Table 2 resulted in fever in 16–18% of subjects after the first dose. This is similar in frequency to the second dose of the mRNA vaccines, which was observed in 14–15.5% of subjects. Fever appeared to decrease after the second dose. In comparison, there was no increase in fever in Gardasil^®^-9 recipients after the second and third doses [47].

The immunogenicity data from AZD1222 are promising [26] and appear to be comparable to those of the mRNA vaccines, although comparisons are difficult due to different assays employed (see Table 3 and accompanying notes).

Cansino’s Ad5-nCoV (Convidecia) vaccine has been in use in China since mid-2020 well before Phase III trials were finished enrolling participants under a special 1-year military authorization (https://www.globaltimes.cn/content/1207517.shtml (accessed on 9 February 2021)). Press release information, released 8 February 2021, indicates a 65.7% vaccine efficacy for Convidecia, based on a study of 30,000 participants out of Pakistan (https://www.bloomberg.com/news/articles/2021-02-08/pakistan-says-cansino-s-covid-vaccine-shows-65-7-efficacy (accessed on 9 February 2021)). The Phase II data in terms of safety, tolerability and immunogenicity [28,29] are adequate and indicative that (see Table 2 and Table 3) the vaccine will be a useful addition to the COVID-19 vaccine armamentarium.

The Gamaleya Sputnik V Phase III data were released 2 February 2021 showing an efficacy rate of 91.6% based on an analysis of 78 COVID-19 cases placebo (62 cases) and vaccine (16 cases) in a 1:3 placebo/active vaccine randomized study (see Figure 1 and Table 1) [19,30]. Earlier published Phase I/II data [30] reveal an adequate safety profile but with elevated pain, fever, headaches in the vaccine group (Table 2) and the induction of a virus-specific antibody response (Table 3).

J&J (Janssen) Ad26.COV2.S vaccine efficacy data from interim press release information (https://www.jnj.com/johnson-johnson-announces-single-shot-janssen-covid-19-vaccine-candidate-met-primary-endpoints-in-interim-analysis-of-its-phase-3-ensemble-trial#:~:text=Phase%203%20ENSEMBLE%20Study%20Safety%20Data&text=Overall%20fever%20rates%20were%209,to%20the%20active%20vaccine%20candidate. (accessed on 9 February 2021)) indicate 66% efficacy across a range of countries where novel SARS-CoV-2 variants are emerging, such as in South Africa (see Figure 1, Table 1 and Section 5 below). Recently published Phase II data [31] and interim Phase III data provide evidence for acceptable safety, tolerability and immunogenicity (Table 2 and Table 3).

### 3.3. Protein-Based Vaccines—Safety/Reactogenicity and Immunogenicity/Efficacy

Complete vaccine efficacy data are eagerly anticipated for this vaccine platform due to strong results pointing to excellent immunogenicity [34,35]. Interim data for the Novavax candidate NVX-CoV2373 points to 89.3% efficacy (https://ir.novavax.com/news-releases/news-release-details/novavax-covid-19-vaccine-demonstrates-893-efficacy-uk-phase-3 (accessed on 9 February 2021)) (see Figure 1 and Table 1) and interesting facts are emerging related to vaccine efficacy against novel SARS-CoV-2 variants (see Section 5 below). This program has encountered significant delays in advancement, especially in the USA for a number of reasons including a high rate of placebo arm Phase III participants dropping out of trials to receive mRNA vaccines [55]. Medicago’s plant-based protein/VLP candidate is lagging well behind in the field of twelve that initially announced Phase III trials.

Both protein/VLP candidate vaccines had an acceptable safety profile in Phase I/II trials, we know that both protein/VLP candidate vaccines are safe [34,35]. The primary adverse events include elevated pain at the injection site, and headache, primarily after the boosting dose (Table 2), which are common with all vaccines. An increase in percentage of subjects with fever was noted for the Medicago vaccine after the second dose, which was also observed for both mRNA vaccines (see Section 3.1). An increase in reactogenicity has been observed after re-vaccination with some vaccines, such as pertussis, varicella and tetanus, but the reasons for this are poorly understood [56].

Table 3 shows the immunogenicity data from earlier Phase II clinical trials. Both protein-based vaccines show strong increases in antiviral immune responses after the second dose. The Medicago vaccine increased antiviral antibody titer and spike protein specific IFN-γ CD4^+^ T cells after the booster dose, but only limited data were available for the Novavax vaccine at time of writing. Both vaccines show evidence for Th1 biased immune responses, attributed to the powerful adjuvants formulated with these vaccines [34,35].

### 3.4. Inactivated Viral Vaccines—Safety/Reactogenicity and Efficacy/Immunogenicity

There are some early indications that the inactivated viral vaccines possess lower efficacy compared to the mRNA platform vaccines but still with adequate protection marginally exceeding WHO guidelines of 50% efficacy. The studies using Sinovac’s CoronaVac vaccine have been overshadowed by discrepant figures cropping up in interim Phase III analyses from the different countries (ranging from a high of 91.25% in Turkey to a low of 65% in Indonesia) before finally settling on a 50.4% efficacy readout from nearly 10,000 subjects enrolled in Brazil. Sinopharm’s (Beijing) candidate has a 79.3–86% efficacy rate (the latter number coming from a study carried out in the UAE) with Sinopharm’s (Wuhan) somewhat lower at 72.5% (see Table 1). However, the full data sets are required for accurate assessment of efficacy for all three of the vaccines made in China. Efficacy of COVAXIN has not yet been reported as of February 2021 but it has been approved in India, so it is being assigned a >50% efficacy readout (see Figure 1 and Table 1). As for safety, the incidence of pain, headache and fever was low from the inactivated virus platform and not significantly increased compared to placebo-injected recipients (Table 2). Adverse events were much lower than with mRNA or viral vector vaccines.

Some inactivated vaccines induce a Th2 immune response, which has been associated with vaccine associated enhanced respiratory disease (VAERD) in humans and animal models of coronavirus disease [57]. For this reason, there is of interest to know if the immune response induced by the different vaccine platforms are Th1 or Th2 biased [17,22,26,54]. Bharat Biotech’s COVAXIN has used the novel adjuvant Algel-IMDG, which is an imidazoquinoline adsorbed on alum [42]. The Th1/Th2 ratio for this vaccine shows a very strong Th1 bias (Table 3) with a good reactogenicity profile (Table 2). The imidazoquinolines are Toll-like receptor 7/8 agonists [58], and to the best of our knowledge, this is the first time it has been widely used as a vaccine adjuvant in humans.

## 4. Comparative Analysis of Target Product Profiles

**Figure 2:**
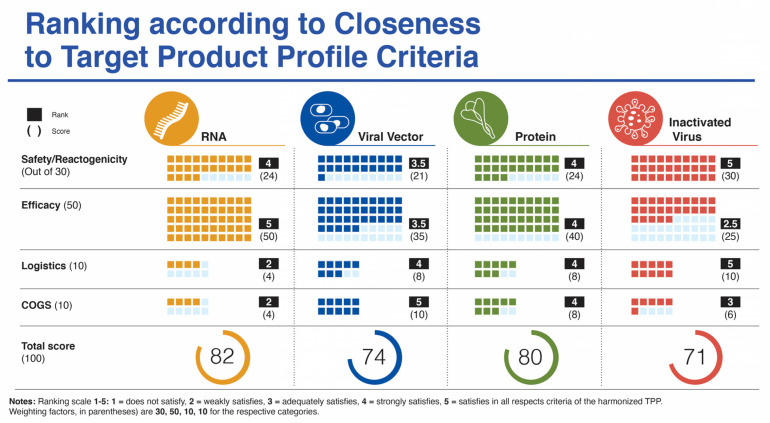
Ranking of four vaccine platforms according to criteria for harmonized target product profile as mentioned in the text.

Supplemental Table 1 lists the target product profile characteristics for a vaccine targeting COVID-19 from four sources. In this section, we focus specifically on five categories: (i) safety/reactogenicity; (ii) efficacy; (iii) dosing regimen; (iv) product stability/storage/supply chain (i.e., logistics); and (v) target price/accessibility (COGS). We start each category section with a “harmonized” TPP version of guidance from CEBR, CEPI, WHO, and our previous publication. A ranking chart compares how well the vaccines match with the harmonized TPP using a scoring system of 1–5 and a weighting parameter for each category to provide a value out of 100 (Figure 2).

### 4.1. Safety/Reactogenicity—A SARS-CoV-2 Vaccine Should Provide a Clear Benefit/Risk Profile Based on 2^+^ Data Sets with at Least 10,000 Subjects across All Age Groups with the Dosing Regimen Intended for Licensure

The minimum data sets and numbers of subjects enrolled and tested have clearly been met by the mRNA platform vaccines with young (16–18 y.o.) to elderly included. The first published datasets from the UK, Brazil, and South Africa Phase III AZ/Ox trials fell short in terms of numbers, especially with the altered dosing regimen [18] but this criteria will be met with additional data emerging [20], and when the numbers from the other three members of this platform are included. The same holds true for the four inactivated virus platform vaccine candidates, which have been tested in many countries (e.g., UAE, Bahrain, Indonesia, Turkey, China, India, etc.) and across demographics to meet the criteria of the harmonized TPP. With the recent release of interim data from the protein/VLP platform, with 15,000 enrolled in UK and 4000+ in South Africa this platform is near to meeting the harmonized TPP guide but would be strengthened by a dataset from Medicago’s vaccine candidate.

In terms of safety/reactogenicity, the inactivated virus platform appears at the top (Ranking 5), while the others rank somewhat below. The viral vector platform scores below the other three platforms (Ranking 3.5) due to some lingering concerns related to paused trials and adverse events, more SAEs reported and the remote possibility for viral vector DNA integration into host cells (Figure 2).

### 4.2. Vaccine Efficacy—A SARS-CoV-2 Vaccine Should Demonstrate at Least 70% Efficacy, with Rapid Onset of Protection (<2 Weeks from Initial Dose), across All Age Groups, from Adolescents to Elderly with Appropriate Evaluation of Humoral and Cellular Immune Responses to Provide a Minimum Serological Correlate of Protection

The clear winners in this category are the mRNA vaccines (Ranking 5) with efficacy in the 95% range and across a spectrum of ages (from age 16 (Moderna) to elderly) and ethnicities, followed by the protein subunit platform (Ranking 4) with the recent data from Novavax revealed on 28 January 2021 with an efficacy of just under 90% in the UK. Next, is the viral vector platform (Ranking 3.5) with mixed efficacy between individual vaccine candidates in this group. The inactivated virus platform ranks lowest for this parameter (Ranking 2.5) based on currently available variable data from trials with Sinopharm and Sinovac vaccine candidates (Figure 2).

### 4.3. Compliance (Relating to Dosing Regimen)—A Sars-Cov-2 Vaccine Should Be a Single Dose during an Outbreak (Optimal). A Two-Dose Regimen Is Acceptable Provided There Is a Short Interval (<28 Days) between Prime and Boost (Minimal)

Since all but 2 of the 12 vaccines in the various platforms analyzed in this study use a two-dose regimen, all have been scored at the same ranking to meet the minimal harmonized TPP expectation for this parameter. Two of the four viral vector platform vaccines use a single dose (CanSino and J&J/Janssen), which could argue for a ranking slightly above the others for this platform. However, since the scores are nearly identical, they are not included in the overall ranking depicted in Figure 2.

### 4.4. Logistics (Product Stability/Storage/Supply Chain)—A SARS-CoV-2 Vaccine Should Have a Shelf Life of at Least 1 Year at −70 °C and Be Stable for at Least 1 Month at Fridge Temperature (4 °C)

Presumably, all platforms meet the harmonized TPP guideline for 1-year stability at −70 °C. However, there are considerable differences in vaccine stability at ordinary refrigeration temperatures between platforms with mRNA candidates possessing burdensome cold-chain requirements. This influences the logistics of vaccine storage and distribution and can lead to spoiled vaccines related to critical timing and handling issues. (Ranking 2). In contrast, the inactivated virus and viral vector platforms score highest (Ranking 5), with long-term stability at fridge temperature and relative ease of distribution. Stability information is not yet available for the protein-based vaccines, but these vaccines are formulated to make virus-like particles (VLPs). Similar VLPs include the Hepatitis B vaccine [59] and the AS03 adjuvanted influenza vaccine [60], both of which are stored at 2 to 8 °C long term.

### 4.5. COGS (Target Price/Accessibility)—A SARS CoV-2 Vaccine Should Possess Capability for Rapid Scale-Up Production and Availability of Sufficient Doses at Cost/Dose That Allows Broad Use, Including in LMIC

**Figure 3:**
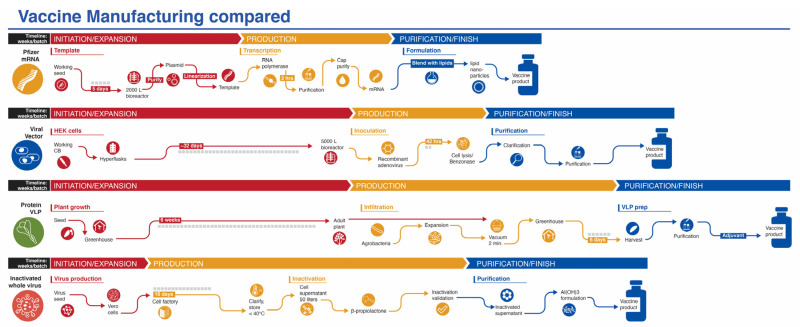
Manufacturing process of vaccines from four platforms covered in this review. The mRNA platform is represented by the Pfizer vaccine. The vaccine development process for the viral vector and inactivated whole virus platforms process are generic, while the protein/VLP platform is represented by the vaccine from Medicago. Timelines are not to scale.

While production of mRNA vaccines can be scaled-up at a reasonable pace, they are currently among the most expensive (https://s3.amazonaws.com/one.org/vaccine-access-test/ONE_VAT_summary_November_EN.pdf (accessed on 9 February 2021)) COVID-19 vaccines, which diminishes their ranking in this category (Ranking 2). The inactivated virus vaccines are relatively easy to produce and are cheap if one considers Bharat Biotech’s COVAXIN. However, there are some indications that the pricing of the vaccines made in China are very high (see three links in notes to Table 1) (Ranking 3). The viral vector vaccines are the cheapest to prepare (Ranking 5), with protein/VLP vaccines somewhat more expensive (Ranking 4). Manufacturing capacity and cost of goods (COGS) analyses give estimated values that match reasonably well with vaccine prices presented in Table 1, affirming that most manufacturers are not making substantial profit on these vaccines during the pandemic. Manufacturing capacity also differs between platforms (Figure 3). Surprisingly, fill/finish is the bottleneck of the manufacturing platforms and contributes significantly to cost and time [61]. The Medicago protein-based platform matches the manufacturing capacity of mRNA vaccines (~10 million doses per month), but each batch takes longer to make (Figure 3).

## 5. Discussion

### 5.1. Successes

Firstly, the global concerted effort to bring a SARS-CoV-2 vaccine targeting COVID-19 into the arms of millions of citizens around the world in less than a year has been a resounding triumph for science in general and for vaccine research and development in particular. The path has not been easy and there have been numerous challenges, obstacles, and significant suffering and death due to the virus worldwide. However, health care workers, front line essential workers, organizations, companies, and countries have rallied around the “global village” concept and the solidarity of cooperation that “we are all in this together” and “this won’t end for anyone until it ends for everyone (https://www.nytimes.com/2020/04/07/opinion/coronavirus-united-states-leadership.html (accessed on 9 February 2021))”. The successes of epidemiology, public health, genetic testing and diagnostics, rapid whole genome viral sequencing and outbreak surveillance should be lauded rather than sifting through the many shortcomings.

Secondly, a novel vaccine platform (mRNA) has been established that will likely alter the course of vaccine development in perpetuity. This will pave the way for the use of the platform in cancer therapeutics and other biomedical applications.

### 5.2. Barriers/Hurdles

Firstly, while we know that antibody levels induced by natural infection with SARS-CoV-2 last several months, we do not know if there will be a requirement for repeat vaccine dosing on an annual (or other timeframe) basis. If repeat booster doses are required, especially related to the viral vector platform, will antibodies be directed to the vector and will this diminish vaccine efficacy?

Secondly, determining an immunological correlate of protection against SARS-CoV-2 is an important objective that still has not been achieved and will be crucial in facilitating future COVID-19 vaccine development and licensing. Since the Pfizer and Moderna vaccines showed efficacy starting at 11 days after the first dose, the opportunity exists to identify a serological correlate of protection. The virus neutralization titer for the Pfizer vaccine was 13 after the first dose, just slightly above the cut-off for the assay, and 316 after the second dose. Likewise, the titer was 149 for the Moderna vaccine (first dose) and 1096 (second dose), respectively. A calculation based on early data shows a nearly significant correlation between vaccine efficacy versus post-vaccination neutralization log-titer (r^2^ = 0.39, *p* = 0.07, see Supplementary Materials Table S3, Figure S1). It is possible that cell-mediated immunity (CMI) is a stronger correlate of protection against COVID-19 disease than antibody-mediated neutralization [62,63], but the failure to demonstrate a significant relationship may be simply due to the different methods used to measure neutralization titer. This calls for a global collaboration to develop standardized assays, as was done for the pneumococcal vaccine [64] and by the WHO for the meningococcal conjugate vaccine (https://apps.who.int/iris/handle/10665/66298 (accessed on 9 February 2021)).

Thirdly, SARS-CoV-2 viral variants are emerging at an alarming rate from around the world [65,66,67,68,69]. Currently, the most discussed viral variants are those that have originated in the UK (known as B.1.1.7), South Africa (B.1.351; also known as 501Y.V2), and Brazil (P.1), respectively (see this resource for a discussion of these and others (https://www.the-scientist.com/news-opinion/a-guide-to-emerging-sars-cov-2-variants-68387?utm_campaign=TS_DAILY_NEWSLETTER_2021&utm_medium=email&_hsmi=108329977&_hsenc=p2ANqtz-9N6VU4j0jsVdUrSG3a3WyIb9L1PLdJGrTihQesrURWRf6Dg143VX51×8TqqgidfNnTOuC1MqOBdMjHQD71SyUkL4UBvA&utm_content=108329977&utm_source=hs_email (accessed on 9 February 2021))). These variants each express a varied assortment of mutations (compared to the original SARS-CoV-2 Wuhan isolates) but all possess the asparagine→tyrosine N501Y mutation that appears to increase the ability of ACE2 to interact with the viral spike receptor-binding domain (RBD). Early indications suggest that the mRNA vaccines are still effective against B.1.1.7 [70,71,72]. Another mutation within the spike protein of variant B.1.351, glutamic acid→lysine E484K, is particularly troubling and this has perhaps led to the reduced efficacy of vaccination in Phase III clinical trials that have taken place in South Africa with the J&J and Novavax vaccines (see notes ^d,e^ in the Table 1 legend). Currently, there is no direct evidence that the variants heighten disease severity but this important point is being investigated. These and other emerging variants possess the potential to derail vaccine efforts to eradicate SARS-CoV-2 viral spread. Moderna has already pre-emptively started production of a new mRNA vaccine candidate targeting B.1.351 and intends to carry out booster vaccinations with this new vaccine [68].

Finally, public perception and compliance in vaccine administration are also very large hurdles to surmount in order to achieve herd immunity in some countries/populations. While not discussed in this review, this is a key point that cannot be overlooked.

### 5.3. Limitations of This Analysis

To date only a few of the twelve vaccine candidates that had announced Phase III trials by mid-November 2020 have published their complete datasets from these trials. Some parts of the analyses have had to rely on partial or incomplete datasets for the Phase III trials or to make use of the Phase II data for some parameters. The rankings presented in Figure 2 are an approximation to the ideal SARS CoV-2 vaccine target product profile with not all fourteen specific criteria evaluated as listed in Supplemental Table S1.

### 5.4. Concluding Remarks

Ten of the twelve vaccines discussed in this article have received some form of authorization for use in different countries around the world as of the end of February 2021. Vaccine rollout has begun in massive campaigns, in Israel, UAE, and across the United States, while the process has been slow throughout Europe, Canada and developing nations. Early success is emerging in Israel [73] where approximately half the population has been vaccinated, with cases of COVID and hospitalization falling dramatically. There has been widespread concern of a two-tier system where rich nations monopolize the expensive, “high-tech” rapidly deployed mRNA vaccines, while other low and middle income countries (LMIC) receive the less-tested (and as-of-yet) incompletely revealed vaccine efficacy candidates. Nonetheless, the successes should be praised and hope should reign that the global pandemic will end soon or at least be held in check so that we can return to a world unhampered by restrictions and to global prosperity.

## Data Availability

All data are contained within the References section and Supplemental Materials.

## Supplementary Materials

The following are available online at https://www.mdpi.com/1999-4915/13/3/418/s1, Figure S1: Clinical efficacy vs neutralization titer (NT) for each vaccine with available data, Table S1: Target product profiles for COVID-19 vaccines, Table S2: Comparison of the formulation and stability of two mRNA vaccine lipid nanoparticles using information from the product monographs, Table S3: Correlation of vaccine clinical efficacy vs post-dose 2 virus neutralization GMT.

## Abbreviations

COVID-19: Coronavirus disease 2019; SARS, Severe Acute Respiratory Syndrome; Ab, antibody; Ad, adenovirus; AZ/Ox, Astra Zeneca/University of Oxford; TPP, target product profile; CEPI, Coalition for Epidemic Preparedness Innovations; dpd, days post-dose; GAVI, GAVI The Vaccine Alliance (formerly Global Alliance for Vaccines and Immunization); WHO, World Health Organization; FDA, United States Food and Drug Administration; EUA, emergency use authorization; ACTIV, Accelerating COVID-19 Therapeutic Interventions and Vaccines; VAERD, vaccine-associated enhanced respiratory disease; LNP, lipid nanoparticle; IFN, interferon; J&J, Johnson & Johnson/Janssen; VLP, virus-like particle; AE, adverse event; SAE, serious adverse event; Th, T helper; CMI, cell-mediated immunity; COGS, cost of goods sold; LMIC, low-middle income countries; HPV, human papilloma virus; i.m., intramuscular; vp, viral particles; y.o., years old; VRBPAC, Vaccines and Related Biological Products Advisory Committee.
