# Supplementary material for: Target Product Profile Analysis of COVID-19 Vaccines in Phase III Clinical Trials and Beyond: An Early 2021 Perspective
Source: Viruses. 2021 Mar 5;13(3):418. doi: 10.3390/v13030418 (PMC7998902; doi:10.3390/v13030418)
Supplement: Supplementary file 1 [file viruses-13-00418-s001.pdf]

Supplementary File

Target Product Profile Analysis of COVID-19 Vaccines in Phase III Clinical Trials and Beyond: An Early 2021 Perspective

Colin D. Funk[1,2*], Craig Laferrière[2], Ali Ardakani [2]

[1]Queen's University, Department of Biomedical and Molecular Sciences, Kingston, ON, K7L 3N6 Canada

[2]Scientific Research Division, Novateur Ventures Inc., Vancouver, BC V6E 3P3 Canada

*** Correspondence:**

Colin D. Funk, funkc@queensu.ca; Craig Laferrière, craig.laferriere@utoronto.ca;
Ali Ardakani, ali@novateur.org

Supplementary Tables S1-S3 and Supplementary Figure S1

Table S1 Target Product Profiles for COVID-19 Vaccines

TPP	CBER (advice)	CEPI (minimal)	WHO (preferred)	Funk et al
**(a)** Indication: Outbreak (OB) and long term (LT).	-Either lab-confirmed COVID-19 or SARS-CoV-2 infection is an acceptable 1º endpoint for a COVID-19 vaccine efficacy trial<br>-Severe COVID-19 should be evaluated as a 2º endpoint.	-For active immunization of persons at-risk for disease to prevent COVID-19 morbidity/mortality.<br>-Risk groups include the elderly, those with underlying chronic diseases, those in contact with patients with confirmed COVID-19, and health care workers.	-For active immunization of persons in the area of an on-going outbreak for the prevention of COVID-19 (OB).<br>-For active immunization of at-risk persons to prevent COVID-19 (LT).	-For use in (current) pandemic response and subsequent wave outbreaks.<br>-For use in routine immunization, supplementary immunization and stockpiling of vaccine against COVID-19.
**(b)**Contraindication	-COVID-19 vaccine trials need not screen for nor exclude participants with history or lab evidence of prior SARS-CoV-2 infection.	-Contraindication in some special populations (e.g. individuals with compromised immune function, pregnant women); acceptable, if other vaccine characteristics favor the vaccine.	-None.	----
**(c)** Target Population	-Enrollment of populations most affected by COVID-19, specifically racial and ethnic minorities.<br>-Adequate representation of elderly individuals and individuals with medical co-morbidities.<br>-Support inclusion of pregnant women and women of child-bearing potential who are not actively avoiding pregnancy in clinical trials	-Focus on healthy adults first, followed by elderly and pediatric populations, excluding pregnant and lactating women.<br>-Countries at risk for COVID-19 transmission.	-For all ages.<br>-Suitable for administration to pregnant and lactating women.	-For all individuals aged 9 months and above (limited information on administration of SARS-CoV-2 vaccine to pregnant or lactating women).<br>-For all countries around the world.
**(d)** Safety/ Reactogenicity	-Studies should include pre-specified criteria for halting based on signals of potential	-Safety and reactogenicity whereby vaccine benefits clearly outweigh safety risks.<br>-Additional pre-clinical data without evidence	-Safety and reactogenicity sufficient to provide a highly favorable benefit/risk profile in the context of observed vaccine	-The safety profile should be based on at least two data sets with preferably at least 10,000

	vaccine-associated ERD.<br>-At least 3000 study participants should be vaccinated with the dosing regimen intended for licensure.	of harmful immune response in animal models (i.e. inducement of virus-enhancing antibodies, eosinophilic lung infiltration).	efficacy; with only mild, transient adverse events related to vaccination and no serious AEs.	subjects over all age groups.
**(e)** Measures of Efficacy	-The primary efficacy endpoint should be at least 50 %, and the statistical success criterion should be that the lower bound of the appropriately α-adjusted confidence interval is >30 %<br>-Studies should include an evaluation of humoral, cellular, and functional immune responses, as appropriate to each of the included COVID-19 antigens.	-At least 70 % protective efficacy against disease caused by COVID-19 in healthy adults.	-At least 70% efficacy (on population basis, with consistent results in the elderly).<br>-Endpoint may be assessed vs. disease, severe disease, and/or shedding/transmission<br>-Rapid onset of protection (less than 2 weeks)(OB).<br>-Rapid onset of protection is less important (LT).	-Phase III clinical studies should include an immunogenicity arm in which serum and blood samples are collected and tested for antibody and cell-mediated immunity.<br>–A minimum serological correlate of protection should be determined based on the short-term and long-term efficacy.
**(f)** Dose Regimen	----	-Two-dose 1º immunization regimen with preference for short interval between doses (max 0-28 days) and with some protection / immune response considered to be protective after the first dose.	-Single-dose primary series (OB).<br>Lower frequency (Yearly or less) of booster doses is preferred (LT).	-Single dose 0.5 ml or less, if injectable. A second boosting dose 4-8 weeks later, if necessary.
**(g)** Duration of Protection	-Follow-up at least one to two years to assess duration of protection and potential for vaccine-associated ERD as immune responses to the vaccine wane.	-Confers protection for at least 6 months without booster vaccination.<br>Duration of protection may be inferred from immune kinetics, as well as documentation of breakthrough cases.	-Confers protection for at least 1 year.	----

**(h)** Route of Administration	----	-Injectable (i.m., s.c., or i.d.) using standard volumes for injection.<br>-Other routes of administration (including those involving electroporation) acceptable, if other vaccine characteristics favor the vaccine in the context of this TPP	-Non-parenteral (syringe/needle or other adjunct equipment-avoiding) is preferred for ease of rapid administration and other logistical issues (OB).<br>-Any route of administration is acceptable (LT).	-Oral (preferred). -Subcutaneous, intradermal, or intramuscular (acceptable).
**(i)** Product Stability, Storage, and Supply Chain Considerations	-The stability and expiry date of the vaccine in its final container, when maintained at the recommended storage temperature, should be demonstrated using final containers from at least three final lots made from different vaccine bulks.	-Shelf life of at least 12 months at up to -70 ºC. Stability of at least 1 month at 2-8 ºC should be demonstrated.<br>-The need for a preservative is determined and any issues are addressed.	-Higher storage temperatures and higher thermostability will greatly enhance vaccine distribution and availability, and are thus strongly preferred.	-Thermostable, not light sensitive. The stability of vaccine potency is durable >2 yrs at room temperature.
**(j)** Co-Administration with other Vaccines	----	-Evidence on safety and immunogenicity when co-administered with influenza vaccines should be considered.	-Stand-alone product (OB)<br>-Potential for coadministration with other vaccines that are typically administered in campaigns preferred (LT).	-The SARS-CoV-2 vaccine should not be administered with any other vaccine or it can be administered concomitantly with influenza vaccine at separate sites.
**(k)** Presentation	----	-Vaccine provided as a liquid or lyophilized product in mono-dose or multi-dose presentations with a maximal dose volume of 1.0 ml.<br>-Multi-dose presentations should be formulated , managed and discarded in compliance with WHO’s multi-dose vial policy.	-Availability of multi-dose presentation is generally preferred for use in campaigns (OB).<br>-Mono-dose or multi-dose presentations are acceptable. Maximum parenteral dose volume: 0.5 mL (LT).<br>-Multi-dose presentations should be formulated, managed and discarded in compliance with WHO’s multi-dose vial policy.	-Single-use or multi-dose vial liquid or lyophilized, stored at room temperature.<br>-Contains SARS-CoV-2 vaccine in sterile liquid (or other format) for direct oral use or in lyophilized format with simple reconstitution at point of use not requiring end-to-end cold chain.

**(l)** Accessibility Pricing target	----	----	-Capability to rapidly scale-up production at cost/dose that allows broad use, including in LMIC (OB).<br>-Availability of sufficient doses at cost/dose that allows broad use, including in LMIC (LT).	-Cost of goods should be reasonable to governments of both high- and low-income nations.
**(m)** Consistent Manufacture	-Data on the manufacture of at least three commercial-scale batches are sufficient to support the validation of the manufacturing process.<br>-This testing can be incorporated into the design of an efficacy trial and does not need to be conducted in a separate study.	-Scalable to produce at least one million doses per month of drug substance	----	----

Table S2. Comparison of the formulation and stability of two mRNA vaccine lipid nanoparticles using information from the Product Monographs

Moderna mRNA-1273 [1]	Pfizer BNT162b2 [2]
**Composition**	
mRNA	mRNA
lipid SM-102<br>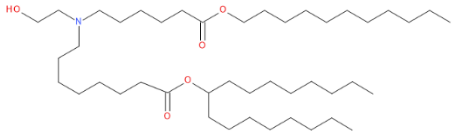<br>heptadecan-9-yl 8-((2-hydroxyethyl)(6-oxo-6-(undecyloxy)hexyl)amino)octanoate) *	ALC-0315<br>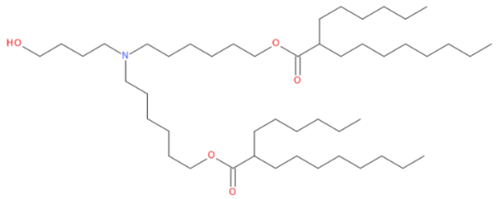<br>((4-hydroxybutyl)azanediyl)bis(hexane-6,1-diyl)bis(2-hexyldecanoate)<br>IUPAC name:<br>6-[6-(2-hexyldecanoyloxy)hexyl-(4-hydroxybutyl)amino]hexyl 2-hexyldecanoate
polyethylene glycol (PEG) 2000 DMG (dimyristoyl glycerol)<br>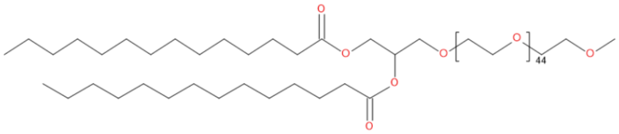	ALC-0159 = 2-[(polyethylene glycol)-2000]-N,N-ditetradecylacetamide<br>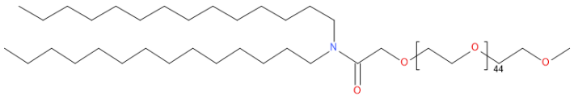
tromethamine $H_2NC(CH_2OH)_3$	potassium chloride
tromethamine hydrochloride	sodium chloride
sodium acetate	dibasic sodium phosphate dihydrate
acetic acid	monobasic potassium phosphate
sucrose	sucrose
cholesterol	cholesterol
1,2-distearoyl-sn-glycero-3-phosphocholine	1,2-distearoyl-sn-glycero-3-phosphocholine
water for injection	water for injection
**Storage and Stability**	
The Moderna COVID-19 Vaccine multiple-dose vials are stored frozen between -25º to -15ºC (-13º to 5ºF). Store in the original carton to protect from light.	Store in the freezer between -80°C to -60°C (-112°F to -76°F). Vials must be kept frozen between -80°C to -60°C (-112°F to -76°F) and protected from light.
Vials can be stored refrigerated between 2° to 8°C (36° to 46°F) for up to 30 days prior to first use.	Defrost in a refrigerator and store [2°C to 8°C (35°F to 46°F)] for up to 5 days (120 hours).
Unpunctured vials may be stored between 8° to 25°C (46° to 77°F) for up to 12 hours.	After dilution, multiple dose vials of Pfizer-BioNTech COVID-19 Vaccine must be stored between 2°C to 25°C (35°F to 77°F) and used within 6 hours from the time of dilution.

1. Monograph. Moderna COVID-19 Vaccine mRNA-1273, December 23, 2020. 2020; https://pdf.hres.ca/dpd_pm/00059386.PDF.
2. Monograph. PFIZER-BIONTECH COVID-19 VACCINE COVID-19 mRNA Vaccine, December 9, 2020. https://pdf.hres.ca/dpd_pm/00059220.PDF.

* Clinical study protocol mRNA-1273-P301, Amendment 6, ModernaTX, Inc., Dec. 23, 2020; accessed on line Jan. 27, 2021

Table S3. Correlation of vaccine clinical efficacy versus post-dose 2 virus neutralization GMT.

Table S3A. Summary of efficacy and immunogenicity data from Tables 1 and 3.

Vaccine	CMI	NT	log NT	Efficacy	+95%CI	-95%CI
Pfizer	0.08	316.1	8.30	95	97.6	90.3
Moderna	0.09	1095.8	10.10	94.1	96.8	89.3
AZ	0.097	128	7	70.4	80.6	54.8
J&J		249	7.96	66.1	74.8	55.01
CanSino	0.01	18.3	4.19	65.7		
Sinovac	0.07	23.8	4.57	50.4		
Novavax	0.18	3906	11.93	89.3		
Sinopharm		282	8.14	79.3		
Sputnik		44.5	5.48	91.6	95.2	85.6

CMI= cell-mediated immunity; GMT = neutralization geometric mean titer from Table 3; log GMT= log base 2 of neutralization GMT; Efficacy from Table 1.

Table S3B. Regression analysis using Microsoft Excel, Data Analysis add-in. Preliminary calculation weighting all results equally.

SUMMARY OUTPUT						
Regression Statistics						
Multiple R	0.62					
R Square	0.39					
Adjusted R Square	0.30					
Standard Error	13.1					
Observations	9					
ANOVA						
	df	*SS*	*MS*	*F*	*Significance F*	
Regression	1	767.0	767.0	4.44	0.073	
Residual	7	1209	172.7			
Total	8	1976				
	Coefficients	*Standard Error*	*t Stat*	*P-value*	*Lower 95%*	*Upper 95%*
Intercept	49.0	14.4	3.39	0.012	14.80	83.1
log NT	3.86	1.83	2.11	0.073	-0.47	8.19

Figure S1 Clinical efficacy vs neutralization titer (NT) for each vaccine with available data.

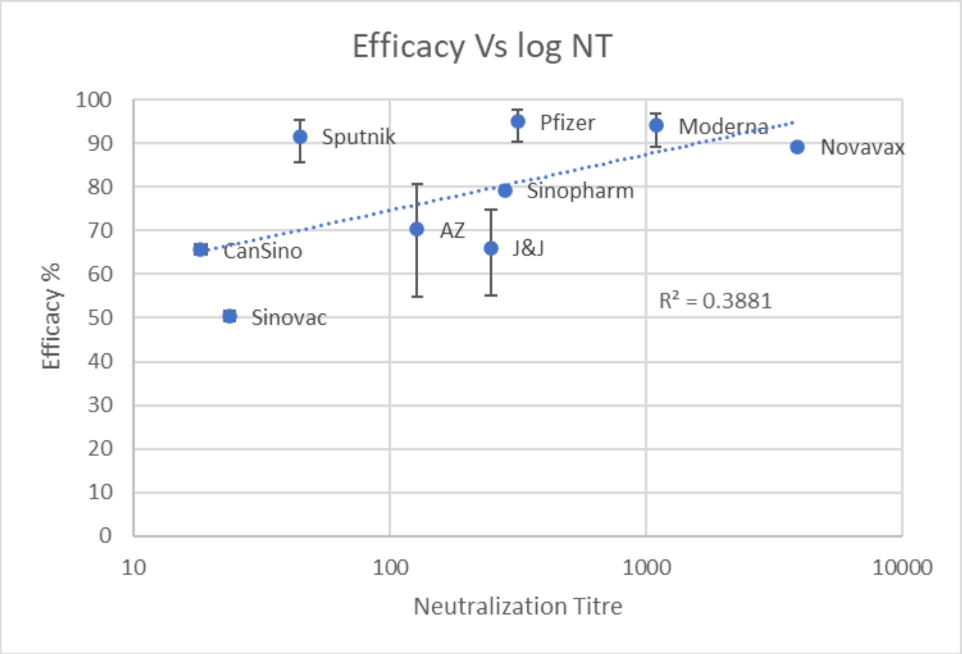

Error bars show 95% CI from published studies. p=0.07 probability slope equals 0. Preliminary calculation weighting all results equally.
